# Resveratrol Pretreatment and Decabromodiphenyl Ethane‐Associated Hepatorenal Changes in a High‐Dose Female Mouse Model: Redox Changes and Ferroptosis‐Associated Markers

**DOI:** 10.1002/fsn3.72495

**Published:** 2026-10-16

**Authors:** Yutong Ji, Chengzhi Liu, Xianhua Wu, Mingxian Shi, Tingjun Hu, Jiakang He, Yingyi Wei, Lei Wang, Meiling Yu

**Affiliations:** ^1^ Guangxi Key Laboratory of Animal Breeding, Disease Control and Prevention, College of Animal Science and Technology Guangxi University Nanning Guangxi China; ^2^ Guangxi Zhuang Autonomous Region Engineering Research Center of Veterinary Biologics Nanning China; ^3^ School of Resources, Environment and Materials Guangxi University Nanning Guangxi China; ^4^ Guangxi Nongken Yongxin Animal Husbandry Group Jinguang Co., Ltd Nanning China; ^5^ Guangxi Health Science College Nanning Guangxi China

**Keywords:** antioxidant‐related transcripts, decabromodiphenyl ethane, ferroptosis‐associated markers, oxidative stress, resveratrol

## Abstract

Decabromodiphenyl ethane (DBDPE) is an emerging brominated flame retardant that has been detected in animal‐derived foods. This exploratory experiment used a high‐dose toxicological challenge rather than a dietary‐exposure scenario. This study evaluated whether resveratrol (Res) pretreatment was associated with differences in selected liver‐ and kidney‐related endpoints in a high‐dose DBDPE mouse model. A total of 54 five‐week‐old specific pathogen‐free female Kunming mice were acclimated for 7 days and allocated into six groups (*n* = 9) by weight‐ranked allocation. Res (25, 50, or 100 mg/kg body weight/day; 100 mg/kg in the Res‐alone group) or vehicle was administered by oral gavage 2 h before DBDPE (500 mg/kg body weight/day) or corn oil, also by gavage, for 28 consecutive days. DBDPE exposure was associated with serum biochemical alterations, descriptive liver and kidney histological changes, redox imbalance, higher iron‐assay values, mitochondrial ultrastructural abnormalities, and changes in ferroptosis‐associated markers. The liver index was 5.06% ± 0.20% in the Control group, 5.89% ± 0.60% in the DBDPE group and 5.17% ± 0.41% in the 100 mg/kg Res pretreatment group; the corresponding kidney indices were 1.02% ± 0.10%, 1.16% ± 0.07% and 1.06% ± 0.03%. Res pretreatment was accompanied by lower values for several DBDPE‐associated endpoints, by changes in Pik3r1, Nfe2l2 and selected antioxidant‐related transcripts, and by tissue‐dependent changes in ferroptosis‐associated markers. These findings provide in vivo associative evidence under the specified experimental conditions and support further evaluation of Res in DBDPE toxicology models; they do not establish ferroptotic cell death, PI3K/Nrf2 pathway activation, or relevance to typical human dietary exposure.

## Graphical abstract

This graphical abstract summarizes the endpoints examined in a 28‐day high‐dose DBDPE exposure model in female mice and the changes associated with resveratrol (Res) pretreatment. DBDPE exposure was accompanied by redox imbalance, higher iron‐assay values, changes in ferroptosis‐associated markers, mitochondrial ultrastructural abnormalities, and hepatorenal changes. Res pretreatment was associated with lower values for several of the measured endpoints. The graphical abstract describes associations among the measured endpoints; it does not establish ferroptotic cell death or PI3K/Nrf2 pathway activation.

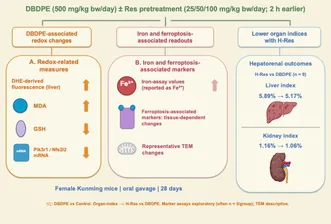

## 1 Introduction

Decabromodiphenyl ethane (DBDPE) is an emerging brominated flame retardant. It has been widely adopted as a major substitute for polybrominated diphenyl ethers (PBDEs) after restrictions on PBDE use and is added to electronic and electrical equipment, plastics, textiles, building materials, and transportation‐related products (Liu et al. 2026). As an additive that is not chemically bound to material matrices, DBDPE is gradually released during production, use, and disposal and accumulates in environmental media (Wang et al. 2023). More importantly from a food‐safety perspective, DBDPE can bioaccumulate in aquatic organisms and transfer along the food chain, and DBDPE residues have been increasingly reported in animal‐derived foods, including eggs, dairy products, meat, and seafood, as well as in drinking water (Li et al. 2024; Liu et al. 2026). Recent risk‐assessment data summarized mean concentrations of 0.0036 ng/kg wet weight (ww) in cow's milk, 24 ng/kg ww in eggs, 18 ng/kg ww in meat, and 45 ng/kg ww in fish and prawns, while concentrations of up to 20.1 ng/kg ww were reported in mussels and oysters (EFSA CONTAM Panel 2026). These occurrence data identify diet as a possible exposure route and motivate toxicological investigation; they do not imply that the exposure level used in the present mouse model is representative of human dietary exposure.

Although DBDPE was once considered a relatively low‐toxicity alternative flame retardant, increasing evidence suggests that its potential biotoxicity should not be overlooked. Previous studies have shown that DBDPE can induce hepatotoxicity, cardiovascular toxicity, thyroid toxicity, and metabolic disorders (Sun et al. 2024; Van Der Schyff et al. 2023). Cellular studies further indicate that DBDPE increases ROS and MDA levels in L‐02 hepatocytes, reduces GSH levels, SOD activity, and mitochondrial membrane potential, and triggers endoplasmic reticulum stress and cellular damage (Jing et al. 2022). These findings suggest that oxidative stress and disruption of subcellular organelle homeostasis may be key molecular events underlying DBDPE‐related health risks after oral exposure.

The liver and kidneys perform important functions in the metabolism, detoxification, transport, and excretion of exogenous chemicals; consequently, they are highly susceptible to damage after exposure to contaminants (Cirino et al. 2023; Lee et al. 2025; Zhu et al. 2024). Given its strong hydrophobicity and bioaccumulation potential, the distribution and metabolism of DBDPE in humans or animals may increase the burden on liver and kidney tissues (Liu et al. 2026). Hepatic and renal dysfunction caused by persistent organic pollutants may also provide mechanistic clues for understanding contaminant‐associated risks to human wellness (Hassan et al. 2021; Yang et al. 2020). Therefore, characterizing the hepatorenal changes associated with DBDPE exposure is important for understanding the potential health implications of contaminant exposure.

Oxidative stress is a central event in tissue injury induced by contaminants. Environmental toxicants can promote reactive oxygen species (ROS) accumulation while weakening enzymatic and non‐enzymatic antioxidant defenses, including superoxide dismutase, catalase, glutathione peroxidase, and reduced glutathione, thereby aggravating lipid peroxidation, inflammatory responses, apoptosis, and structural tissue damage (Bozkurt et al. 2026). Evidence from a cadmium‐associated lung adenocarcinoma model further indicates that persistent contaminant exposure can disrupt extracellular‐matrix remodeling and vascular/immune homeostasis, whereas oxyresveratrol can partially reverse these alterations (Isıyel et al. 2025). When ROS are overproduced and antioxidant defenses are insufficient, redox homeostasis is disrupted, leading to lipid peroxidation, mitochondrial damage, and regulated cell death (Jing et al. 2022). In experimental doxorubicin‐induced cardiac injury, oxidative damage was accompanied by increased malondialdehyde levels, glutathione depletion, and dysregulated antioxidant enzyme activity, whereas esculetin treatment partially restored redox homeostasis (Demir et al. 2026). In recent years, ferroptosis, a form of regulated cell death characterized by iron‐dependent lipid peroxidation, has been recognized as an important mechanism in tissue injury induced by food‐chain and environmental chemical stressors (Sun et al. 2026). Studies on co‐exposure to DBDPE and other contaminants suggest that oxidative stress, ferroptosis, and inflammatory responses may jointly contribute to hepatocyte injury (Shi et al. 2024). Because iron handling, lipid peroxidation, and mitochondrial function are relevant to both contaminant toxicity and regulated cell‐death pathways, their co‐occurrence can motivate investigation of a possible ferroptotic mechanism but cannot by itself establish that mechanism or a temporal sequence from oxidative stress to organ injury.

Resveratrol (Res) is a natural polyphenolic compound widely found in grapes, peanuts, Japanese knotweed, and various medicinal and edible plants (Özdemir and Demir 2025). It shows multiple biological activities, including antioxidant, anti‐inflammatory, lipid‐regulating, mitochondrial protective, and cell death‐modulating effects (Chimento et al. 2025; Rauf et al. 2018; Xia et al. 2017). Because Res is a food‐derived bioactive compound, its effects in contaminant exposure models are of direct interest to food science and human wellness, and purified food‐derived bioactives are widely tested against high‐dose toxic challenges in rodent models (Alkully et al. 2025). Res‐related oxidative‐stress and ferroptosis‐associated endpoints have likewise been examined in this context (Babaeenezhad et al. 2025). Previous studies have shown that Res can modulate lipid peroxidation, iron handling and Nrf2‐related signaling, and it has shown protective effects in various tissue injury models (Hu et al. 2025; Wang et al. 2024; Wang et al. 2025; Yang et al. 2025). In the present study, Res was administered as a purified food‐derived bioactive at defined doses; the doses tested are not proposed as achievable through ordinary dietary intake of Res‐containing foods, and no dietary recommendation is made.

Available DBDPE studies have largely addressed environmental occurrence, single‐organ toxicity, or in vitro mechanisms, leaving limited in vivo information on concurrent liver and kidney responses to repeated oral DBDPE exposure and on how Res pretreatment relates to those responses. The specific contribution of the present study is the combined assessment of hepatorenal outcomes, redox‐related measures, mitochondrial morphology, iron levels, and ferroptosis‐associated markers in one female‐mouse exposure model. Dietary and naturally occurring antioxidants have shown protective potential against related brominated flame retardants; for example, the flavonoid troxerutin alleviated BDE‐47‐induced renal injury by strengthening Nrf2‐mediated antioxidant defense, and N‐acetylcysteine attenuated BDE‐209‐induced cellular oxidative damage (Shan et al. 2017; Zhang et al. 2025). For DBDPE itself, however, the few available rescue studies have relied on pharmacological or molecular tools in vitro, such as ferrostatin‐1 in co‐exposed fish hepatocytes and NAT10 inhibition in placental trophoblasts, rather than food‐derived dietary interventions in vivo (Xie et al. 2025). The present study therefore used Kunming mice to establish a repeated oral DBDPE exposure model with Res pretreatment, and examined whether Res pretreatment was accompanied by differences in hepatorenal outcomes, oxidative‐stress‐related measures, iron‐assay values and ferroptosis‐associated markers.

## 2 Materials and Methods

### 2.1 Main Reagents and Materials

Decabromodiphenyl ethane (DBDPE, 96% purity) and resveratrol (Res, 99.9% purity) were purchased from Shanghai Macklin Biochemical Co. Ltd. Assay kits were purchased from Nanjing Jiancheng Bioengineering Institute (Nanjing, China) and were used according to the manufacturers' instructions. Serum biochemical assays used kits for γ‐glutamyltransferase (γ‐GT, C017‐2), alkaline phosphatase (AKP, A059‐2), albumin (ALB, A028‐2), creatinine (CRE, C011‐2), uric acid (UA, C012‐2), triglycerides (TG, A110‐1), total cholesterol (T‐CHO, A111‐1) and lactate dehydrogenase (LDH, A020‐2). Oxidative‐stress markers used kits for malondialdehyde (MDA, A003‐1), reduced glutathione (GSH, A006‐2), glutathione peroxidase (GSH‐Px, A005‐2), glutathione S‐transferase (GSH‐ST, A004‐1‐1), superoxide dismutase (SOD, A001‐3), catalase (CAT, A007‐1) and total nitric oxide synthase (T‐NOS, A014‐2‐2). Iron in serum and tissue was measured with an iron assay kit (A039‐1‐1). Total protein was measured with a Coomassie brilliant blue kit (A045‐2). Reagents for RNA extraction were obtained from Vazyme Biotech Co. Ltd. (Nanjing, China), and reverse‐transcription and real‐time quantitative PCR reagents were obtained from commercial kits supplied by Sparkjade Biotech Co. Ltd. (Shandong, China). Rabbit primary antibodies against GPX4, ACSL4/FACL4, and transferrin receptor 1 (TFR1/CD71), as well as horseradish peroxidase‐conjugated goat anti‐rabbit IgG, were purchased from Shanghai BoWan Biotechnology Co. Ltd. (recorded lot nos. T56959F, H681704058, T510198F, and F300405, respectively). A rabbit anti‐β‐actin polyclonal antibody (Wuhan Servicebio Biotechnology Co. Ltd.; lot no. AC220730011) was used as the loading control. All other reagents were of analytical grade.

### 2.2 Animals and Experimental Design

A total of 54 five‐week‐old SPF‐grade female Kunming mice, weighing 28–32 g, were purchased from Changsha Tianqin Biotechnology Co. Ltd. (Laboratory Animal Production License No. SCXK (Xiang) 2022‐0011). After arrival, the mice were housed in an SPF animal facility with ad libitum access to food and water, maintained at 24°C–26°C with a relative humidity of 50%–60% under a controlled 12‐h light/12‐h dark cycle, and acclimated for 7 days. Mice were housed with corn‐cob bedding, with ad libitum access to water and to a standard maintenance diet purchased from the same supplier as the animals (Changsha Tianqin Biotechnology Co. Ltd.). No additional environmental enrichment was provided. During the 7‐day acclimation period the 54 mice were housed nine per cage in six large cages (375 × 270 × 170 mm). After allocation, each treatment group was divided into three replicate cages of three mice housed in standard mouse cages (380 × 178 × 139 mm) (three replicate cages per group, 18 cages in total), each cage containing animals of a single treatment group only. The 18 cages were held at fixed positions on the same rack in the same room, and these positions were maintained throughout the 28‐day dosing period. Because the intervention was administered to individual animals by oral gavage rather than at the cage level, the individual animal was treated as the experimental unit; cage was not treated as an experimental unit. The cages were nested within treatment groups, and the implications for the statistical analysis are addressed in Section 4. All animal procedures were conducted in accordance with the Regulations on the Management of Laboratory Animals and were approved by the Animal Use Committee of Guangxi University (GXU‐2021‐1004).

After acclimation, the 54 female mice were ranked in ascending order of initial body weight. Consecutive positions in the ranking were assigned to successive groups in a fixed cyclic order: the first animal to the Control group, the second to the DBDPE group, the third to the L‐Res group, the fourth to the M‐Res group, the fifth to the H‐Res group, and the sixth to the Res‐alone group. This cycle was repeated through all 54 animals, so that the body‐weight range was distributed evenly across the groups. This was a weight‐ranked systematic allocation and not a random‐sequence procedure. Allocation was performed by one of the authors; the investigators who carried out the histological evaluation, DHE‐fluorescence quantification, Western blot densitometry and statistical analysis were blinded to group allocation (Section 2.11). Because the allocation order followed directly from the body‐weight ranking and a fixed cyclic rule, it was fully determined by the ranking and there was no random sequence to conceal; no separate allocation‐concealment procedure was used. The groups were the Control group, DBDPE group, low‐dose Res pretreatment group (L‐Res, 25 mg/kg bw/day), medium‐dose Res pretreatment group (M‐Res, 50 mg/kg bw/day), high‐dose Res pretreatment group (H‐Res, 100 mg/kg bw/day), and Res‐alone group (Res, 100 mg/kg bw/day). The DBDPE dose was 500 mg/kg bw/day, administered by oral gavage. The 500 mg/kg bw/day dose corresponds to the highest dose of our earlier mouse dose‐range study, and the low and medium doses of that study (5 and 50 mg/kg bw/day) were obtained by dividing it by 100 and by 10, respectively. The exposure model follows our previous dose‐range study in Kunming mice, in which DBDPE was administered orally in corn oil at 5, 50 and 500 mg/kg bw/day for 60 consecutive days to male mice. In that study, 500 mg/kg bw/day was associated with an increased liver index, hepatic steatosis and necrosis on histology, and an increased serum uric acid concentration. The kidney index was decreased at 50 mg/kg bw/day, and renal interstitial deposits were observed at 500 mg/kg bw/day. The present experiment adopted the highest dose of that range (500 mg/kg bw/day) to maximize the likelihood of a detectable hepatorenal response, and shortened the exposure to 28 days in female mice receiving Res pretreatment. The corn oil vehicle and the 28‐day duration also follow a published 28‐day oral study in male rats (Jing et al. 2019). The Res doses of 25, 50, and 100 mg/kg body weight/day were selected from dose ranges used in published rodent studies of resveratrol (Shahcheraghi et al. 2023; Wang et al. 2022). In particular, Wang et al. (2022) administered Res by gavage to mice at 25, 50 and 100 mg/kg using the same low‐, medium‐ and high‐dose designations, in a study of iron overload and hepatic iron homeostasis. These doses were designated as the low‐, medium‐ and high‐dose levels, respectively. Doses for each group were calculated according to weekly body weight, with a gavage volume of 0.1 mL per 10 g bw. Group size was fixed in the animal ethics protocol approved by the Animal Use Committee of Guangxi University (GXU‐2021‐1004), which specified 54 animals in six groups of nine. This followed the standard practice for 28‐day repeated‐dose oral gavage studies in rodents. No prospective power calculation was performed. For several terminal assays the number of animals analyzed was three per group. Transmission electron microscopy, Oil Red O staining on frozen sections and RNA extraction each require a separate portion of tissue, and the tissue obtainable from a single animal was not sufficient for the complete assay panel. Total serum volume also limited the number of serum assays. Tissue was collected from all nine animals per group, and three samples per group were selected at random for these assays.

The L‐Res, M‐Res, H‐Res, and Res‐alone groups received the corresponding doses of Res by oral gavage, whereas the Control and DBDPE groups received an equal volume of 4% (v/v) DMSO in saline. Res was freshly prepared each day immediately before dosing and was administered only after complete dissolution. Preparation and administration were completed promptly to minimize light exposure. The freshly prepared Res formulation was administered by oral gavage without storage or reuse. Two hours later, the DBDPE, L‐Res, M‐Res, and H‐Res groups were administered DBDPE at 500 mg/kg bw/day, whereas the Control and Res groups received an equal volume of corn oil. Corn oil was selected as the DBDPE vehicle based on previous oral‐exposure studies in rodents (Jing et al. 2019). The DBDPE–corn oil preparation was thoroughly mixed before administration and remixed immediately before withdrawal of each gavage dose. No visible sediment or precipitation was observed after mixing. DBDPE is described as dispersed in corn oil, and the administered doses are reported as nominal. All animals received two daily oral gavage administrations separated by 2 h for 28 consecutive days, as illustrated in Scheme 1 and summarized in Table S1. All animals were handled individually twice daily for gavage administration throughout the 28‐day dosing period. Clinical signs were assessed at each handling by visual inspection of mental state and activity, coat condition and general condition; body weight was recorded weekly as an additional monitoring measure. No formal clinical scoring system was used; findings were recorded as present or absent at each handling and were not entered on a structured clinical record form. No deaths and no overt abnormal clinical signs were observed in any group.

**SCHEME 1:**
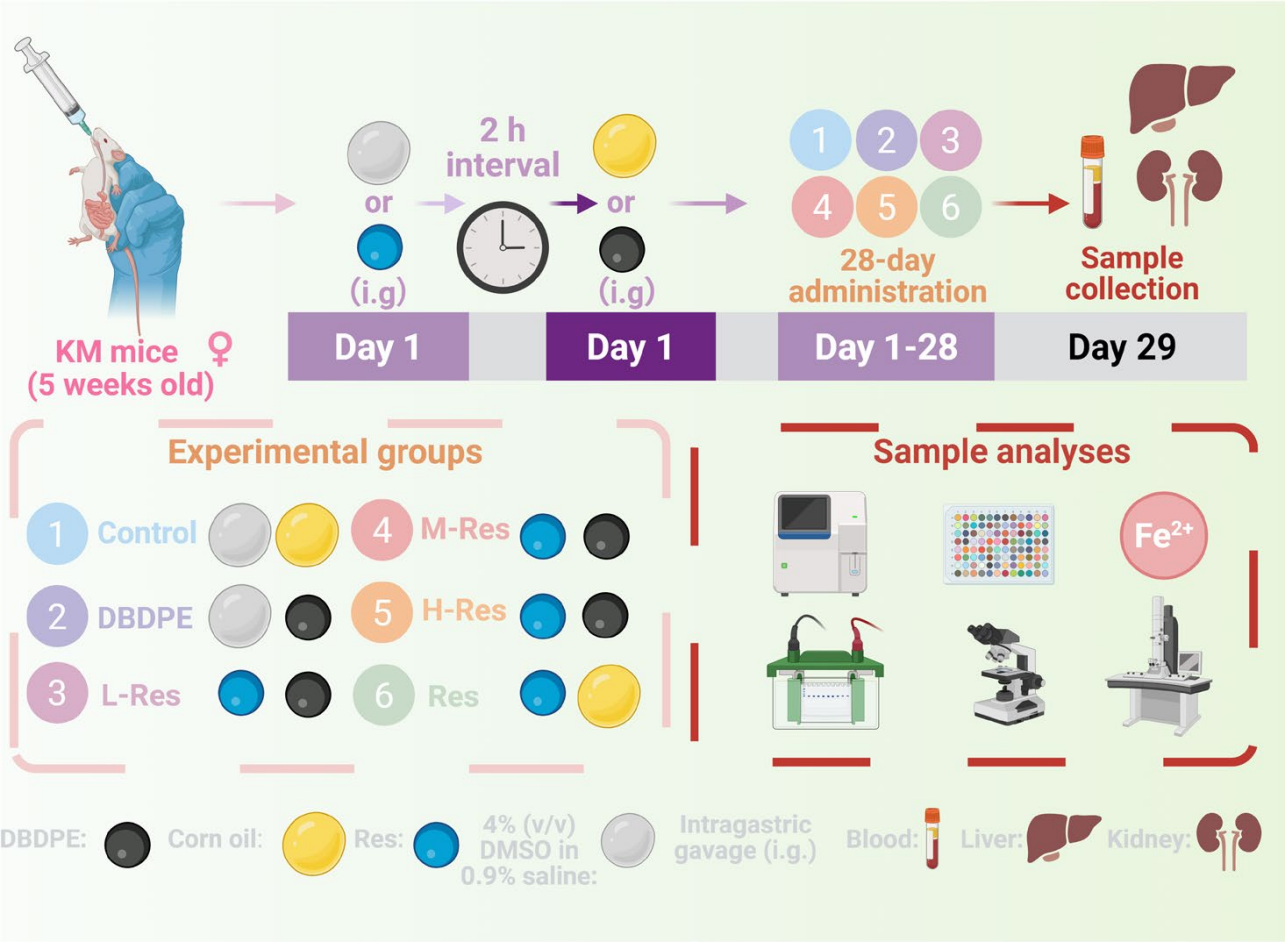
Experimental design for DBDPE exposure and Res pretreatment. After a 7‐day acclimation period, 54 five‐week‐old SPF‐grade female Kunming mice were allocated to six groups (*n* = 9 per group) by weight‐ranked allocation (Section 2.2): Control, DBDPE, low‐dose Res pretreatment (L‐Res), medium‐dose Res pretreatment (M‐Res), high‐dose Res pretreatment (H‐Res), and Res alone. Mice in the L‐Res, M‐Res, H‐Res, and Res‐alone groups received Res by oral gavage at 25, 50, 100, and 100 mg/kg body weight/day, respectively, whereas mice in the Control and DBDPE groups received the corresponding vehicle, 4% (v/v) DMSO in 0.9% saline. Two hours later, mice in the DBDPE, L‐Res, M‐Res, and H‐Res groups received DBDPE by oral gavage at 500 mg/kg body weight/day, whereas mice in the Control and Res‐alone groups received an equal volume of corn oil. This Res pretreatment and DBDPE exposure regimen was repeated once daily for 28 consecutive days. Blood, liver, and kidney samples were collected 24 h after the final administration for serum biochemical analysis, descriptive histological examination, oxidative stress assays, RT‐qPCR, Fe^2+^ measurement, transmission electron microscopy, and Western blot analysis. DBDPE, decabromodiphenyl ethane; Res, resveratrol.

### 2.3 Sample Collection and Organ Index Measurement

At 24 h after the final administration, mice that had been fasted for 12 h with free access to water were subjected to sample collection. The terminal body weight used for organ‐index calculation was the body weight recorded on Day 28 before the fasting period. Sample collection was conducted between 08:00 and 11:00. To minimize potential effects of sampling time and processing order, animals from the different experimental groups were processed in an alternating, group‐balanced sequence. Mice were anesthetized with inhaled isoflurane, and blood was collected within 3 min after an adequate surgical plane of anesthesia was reached. After blood collection, the mice were euthanized by cervical dislocation under anesthesia.

Body weight was recorded weekly throughout the 28‐day experimental period and is reported as group values at each weekly time point. Treatment, time and their interaction were not assessed in a repeated‐measures or mixed‐effects model, so no effect on the trajectory of weight gain is inferred beyond the weekly values.

Blood samples were allowed to clot at room temperature and then centrifuged at 4°C using a high‐speed refrigerated benchtop centrifuge (5430R, Eppendorf, Germany) to obtain serum, which was stored at −80°C for subsequent biochemical, oxidative stress‐related, and Fe^2+^ assays.

The liver and kidneys were rapidly excised, rinsed with saline, and blotted dry. Organs were weighed using a microbalance (EL204, Mettler Toledo, Switzerland), and organ indices were calculated. Organ indices are ratios of organ weight to body weight and may change through either the numerator or the denominator. They are the conventional normalized measure of relative organ size in this type of study and are therefore reported as relative measures only. A portion of liver and kidney tissues was fixed in 4% paraformaldehyde for histopathological examination, another portion was fixed in 2.5% glutaraldehyde for transmission electron microscopy, and the remaining tissues were snap‐frozen in liquid nitrogen and stored at −80°C for oxidative stress assays, RT‐qPCR, Fe^2+^ measurement, and Western blot analysis.




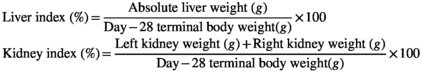




### 2.4 Serum Biochemical Parameter Assays

Serum markers related to liver and kidney function and metabolism included γ‐glutamyltransferase (γ‐GT), alkaline phosphatase (AKP), albumin (ALB), creatinine (CRE), uric acid (UA), triglycerides (TG), total cholesterol (T‐CHO), and lactate dehydrogenase (LDH). All assays were performed according to the manufacturers' instructions. Absorbance was measured using a microplate reader (EnSpire, PerkinElmer, USA) or a UV–visible spectrophotometer (UV‐1800, Shimadzu (Suzhou) Co. Ltd., China), and enzyme activities or concentrations were calculated according to the kit formulas.

### 2.5 Histopathological Examination and Oil Red O Staining

Fixed liver and kidney tissues were dehydrated using an automatic tissue dehydrator (ZT‐12M, Xiaogan Yaguang Medical Electronic Technology Co. Ltd.) and embedded in paraffin using a tissue paraffin embedding machine (YB‐6LF, Xiaogan Yaguang Medical Electronic Technology Co. Ltd.). The embedded tissues were sectioned into 4‐μm serial sections using a rotary microtome (RM2245, Leica Instruments (Shanghai) Co. Ltd.). After spreading and drying on a tissue spreader‐dryer (YT‐7FB, Xiaogan Yaguang Medical Electronic Technology Co. Ltd.), the sections were stained with hematoxylin and eosin (H&E). Histological features in liver and kidney tissues, including cellular arrangement, inflammatory cell infiltration, vacuolar changes, focal necrosis‐like areas, vascular congestion, and renal tubular changes, were observed using a biological microscope (ML51, Guangzhou Mingmei Optoelectronic Technology Co. Ltd.). No semiquantitative or quantitative histopathological scoring system was applied; therefore, the histological findings were interpreted descriptively. Image annotations identify morphological features in the displayed fields; they do not quantify lesion severity or remove observer‐dependent interpretation, and no treatment‐efficacy conclusion is drawn from the representative images. Histological observations were made on representative sections; the number of animals examined per group was not recorded separately from the number of images presented, and no quantitative scoring or morphometric measurement was performed.

To evaluate hepatic lipid deposition, frozen liver tissues were embedded in OCT compound and sectioned into 10‐μm cryosections, followed by Oil Red O staining. After staining, lipid droplet distribution and accumulation in hepatocytes were observed using a biological microscope (ML51, Guangzhou Mingmei Optoelectronic Technology Co. Ltd.).

### 2.6 Detection of Oxidative Stress‐Related Markers and DHE Fluorescence Analysis

Liver and kidney tissues were mixed with prechilled saline at a 1:9 (w/v) ratio, and tissue homogenates were prepared using a high‐speed tissue homogenizer (KZ‐II, Wuhan Servicebio Biotechnology Co. Ltd.). The homogenates were centrifuged in a high‐speed refrigerated benchtop centrifuge (5430R, Eppendorf AG, Germany). The supernatants were used to determine total protein (TP) concentration, malondialdehyde (MDA) content, reduced glutathione (GSH) content, and the activities of glutathione peroxidase (GSH‐Px), glutathione S‐transferase (GSH‐ST), superoxide dismutase (SOD) and catalase (CAT). Serum MDA content and GSH‐ST, GSH‐Px, SOD, and total nitric oxide synthase (TNOS) activities were determined according to the manufacturers' instructions. NOS catalyzes the production of nitric oxide, and alterations in total NOS activity may reflect changes in nitric oxide‐related redox and nitrosative responses. All absorbance measurements were performed using a microplate reader (EnSpire, PerkinElmer, USA) or a UV–visible spectrophotometer (UV‐1800, Shimadzu (Suzhou) Co. Ltd.), and the corresponding concentrations, contents, or enzyme activities were calculated according to the kit formulas.

DHE‐derived fluorescence in liver tissue was assessed using dihydroethidium probe staining. Frozen liver sections were incubated with probe staining solution at 37°C in the dark for 30 min, and fluorescence images were acquired using a fluorescence inverted microscope (TS2‐FL, Nikon (China) Co. Ltd.). At least five random fields were selected from each section, and fluorescence intensity was quantified using ImageJ software. Three independent animals were analyzed per group (*n* = 3); the fields and sections examined are technical measurements and were not counted as additional biological replicates. The signal was treated as an indirect, semiquantitative redox‐associated measure rather than a species‐specific measurement of ROS or superoxide.

### 2.7 RT‐qPCR Assay

RT‐qPCR analysis was performed with reference to previously described methods, with minor modifications (Karağaç et al. 2024). Three independent liver samples and three independent kidney samples from different animals were analyzed per experimental group (*n* = 3). Total RNA was extracted from the tissue samples using the TRIzol method. RNA concentration and purity were determined using a UV–visible spectrophotometer (UV‐1800, Shimadzu, Suzhou, China), and cDNA was synthesized according to the reverse‐transcription kit instructions.

Real‐time quantitative PCR (RT‐qPCR) was performed using the SYBR Green method on a LightCycler 96 real‐time PCR system (Roche, Switzerland). The transcripts measured were Pik3r1, Nfe2l2, Sod2, Cat and Gpx1, and ferroptosis‐associated genes included Tfrc, Fth1, Slc7a11, Gpx4, and Acsl4. Actb was used as the internal control, and relative gene expression was calculated using the 2^−ΔΔCt^ method. The qPCR reaction system and amplification conditions followed the manufacturer's instructions. Each biological sample was analyzed in three technical PCR replicates; technical replication does not increase the biological sample size (*n* = 3 per group for each tissue). Melting curve analysis was performed to confirm amplification specificity. Primer sequences are listed in Tables S2 and S3.

### 2.8 Fe^2+^ Content Assay

Commercial iron assay kits were used to determine Fe^2+^ levels in serum, liver, and kidney tissues. Liver and kidney tissues were homogenized according to the kit instructions using a high‐speed tissue grinder (KZ‐II, Wuhan Servicebio Technology Co. Ltd., China), followed by centrifugation using a high‐speed refrigerated benchtop centrifuge (5430R, Eppendorf, Germany). Absorbance was measured using a microplate reader (EnSpire, PerkinElmer, USA) or a UV–visible spectrophotometer (UV‐1800, Shimadzu (Suzhou) Co. Ltd., China). Serum Fe^2+^ levels were expressed as μmol/L, whereas liver and kidney Fe^2+^ levels were expressed as μmol/g protein. The Fe^2+^ designation refers to the commercial assay output; selectivity for ferrous iron over total assay‐accessible iron was not established, and the results are therefore interpreted as assay‐accessible iron readouts rather than as a specific measure of the ferrous iron pool. The three samples analyzed per group were obtained from three different animals, and no sample pooling was used.

### 2.9 Transmission Electron Microscopy Observation

Approximately 1 mm^3^ of liver and kidney tissue was fixed in 2.5% glutaraldehyde, followed by PBS rinsing, osmium tetroxide postfixation, gradient ethanol dehydration, resin embedding, and ultrathin sectioning. The sections were double‐stained with uranyl acetate and lead citrate. Mitochondrial ultrastructure in liver and kidney tissues was observed using a transmission electron microscope (HT7800, Hitachi, Japan) at an accelerating voltage of 80 kV. Mitochondrial membrane appearance, cristae structure, electron density, and other morphological features were observed qualitatively. No ultrastructural scoring system or quantitative morphometric analysis was applied.

### 2.10 Western Blot Analysis

Liver and kidney tissues were lysed in RIPA buffer and homogenized using a high‐speed tissue homogenizer (KZ‐II, Wuhan Servicebio Technology Co. Ltd., China). The homogenates were centrifuged using a high‐speed refrigerated benchtop centrifuge (5430R, Eppendorf, Germany), and the supernatants were collected for protein quantification. Protein concentrations were determined using the BCA method with a microplate reader (EnSpire, PerkinElmer, USA). After adjustment to equal concentrations, protein samples were mixed with loading buffer and denatured by boiling.

The protein abundance of glutathione peroxidase 4 (GPX4), acyl‐CoA synthetase long‐chain family member 4 (ACSL4), and transferrin receptor 1 (TFR1; encoded by the mouse Tfrc gene) was determined by Western blotting. Rabbit primary antibodies against GPX4, ACSL4/FACL4, and TFR1/CD71 were used at a dilution of 1:2000. β‐actin was used as the loading control. The expected molecular weights were approximately 17 kDa for GPX4, 79 kDa for ACSL4, 90 kDa for TFR1, and 42 kDa for β‐actin. Protein samples were separated by SDS‐PAGE using a standard electrophoresis power supply (PowerPac, Bio‐Rad, USA). Proteins were then transferred to PVDF membranes, blocked with 5% nonfat dry milk, and incubated overnight at 4°C with primary antibodies against GPX4, TFR1, ACSL4, and β‐actin, followed by incubation with HRP‐conjugated secondary antibodies. Signals were detected using ECL chemiluminescence, and band images were captured using an ImageQuant imaging system (LAS 500, GE, USA). Band intensities were analyzed using ImageJ software, and relative protein expression was expressed as the ratio of target protein intensity to β‐actin intensity. Three independent animals were analyzed per group (*n* = 3). The blots shown in Figure 6 are cropped representative bands. Stability of β‐actin across treatment groups was not separately validated.

### 2.11 Statistical Analysis

Group allocation was known to the person who ranked and allocated the animals and to the person who administered the daily gavages. Tissue samples, histological sections, DHE‐fluorescence images, Western blot images and datasets were labeled with coded identifiers before assessment, and the histological evaluation, DHE‐fluorescence quantification, Western blot densitometry and statistical analysis were performed by investigators blinded to group allocation. Group identities were revealed only after completion of the analyses. All quantitative data are presented as mean ± SD, and error bars represent SD. Statistical analysis was performed using SPSS 26.0. Two multiplicity approaches were used and are reported separately. For all endpoints, differences among groups were analyzed by one‐way ANOVA followed by Duncan's multiple comparison test, applied within each experimental endpoint. Different lowercase letters indicate differences at *p* < 0.05 and different uppercase letters indicate differences at *p* < 0.01. No correction was applied across endpoints for these within‐endpoint comparisons. The primary family comprises the comparison of the highest Res dose tested (100 mg/kg) with the DBDPE group for the liver index and the kidney index. For these two comparisons, the mean differences, two‐sided 97.5% confidence intervals and Bonferroni‐adjusted *p* values were obtained from the statistical analysis output, with multiplicity controlled across the two comparisons by the Bonferroni procedure. Normality and homogeneity of variance were checked before the analysis in SPSS 26.0; homogeneity of variance was assessed using Levene's test. The reported estimates rest on those assumptions. Post hoc power calculations were not performed. This primary/exploratory division is based on the directness of each endpoint to the study question and on the adequacy of the group size, not on the statistical significance of the observed differences; no prospective statistical analysis plan was prepared. Graphs were generated using GraphPad Prism 9.0 and Origin 2024.

## 3 Results

### 3.1 Res Pretreatment and DBDPE‐Associated Organ‐Index and Serum Biochemical Changes in Mice

Weekly body weights are shown in Figure 1A and are reported as group values at each weekly time point. Treatment, time and their interaction were not assessed in a longitudinal model, so no effect on the trajectory of weight gain is inferred beyond the weekly values. The liver and kidney indices were 5.06% ±0.20% and 1.02% ± 0.10% in the Control group, 5.89% ± 0.60% and 1.16% ± 0.07% in the DBDPE group, and 5.17% ± 0.41% and 1.06% ± 0.03% in the H‐Res group, respectively (Figure 1B). These group differences describe lower organ‐index values in the H‐Res group than in the DBDPE group. The primary comparison is shown in Figure 1B: the liver index was lower in the H‐Res group than in the DBDPE group (mean difference −0.72%, 97.5% CI −1.32 to −0.12, Bonferroni‐adjusted *p* = 0.018) and the kidney index was also lower (−0.10%, 97.5% CI −0.16 to −0.04, Bonferroni‐adjusted *p* = 0.002), with multiplicity across the two primary outcomes controlled by the Bonferroni procedure.

**FIGURE 1:**
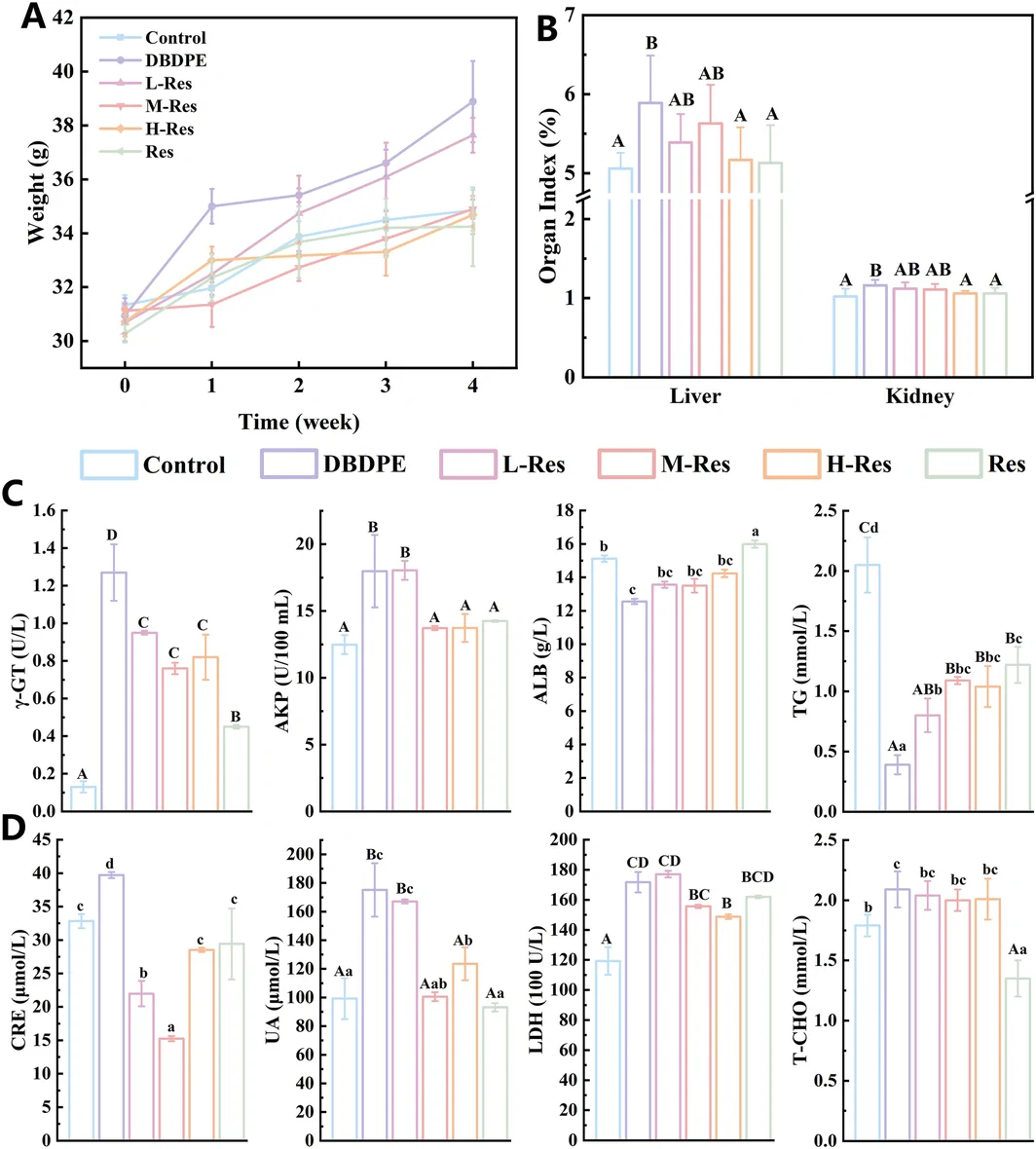
Effects of DBDPE exposure and Res pretreatment on body weight, organ indices, and serum biochemical parameters in female mice. (A) Changes in body weight during the 28‐day experimental period (*n* = 9). (B) Liver and kidney indices (*n* = 9). (C) Serum biochemical markers related to hepatobiliary status and lipid metabolism, including γ‐GT, AKP, ALB, and TG (*n* = 3). (D) Renal‐associated and nonspecific serum biochemical markers, including CRE, UA, LDH, and T‐CHO (*n* = 3).

Serum biochemical analysis showed that DBDPE exposure altered several parameters associated with hepatobiliary status, protein and lipid metabolism, renal‐related biochemical changes, and general tissue injury (Figure 1C,D). Compared with the Control group, the DBDPE group showed higher serum γ‐GT, AKP, CRE, UA, LDH and T‐CHO values and lower ALB and TG values (*p* < 0.05 or *p* < 0.01). Values in the Res pretreatment groups differed from those in the DBDPE group to varying degrees. In particular, γ‐GT, CRE, and UA values were lower and TG and ALB values were higher in one or more Res pretreatment groups than in the DBDPE group. These results describe changes in the measured serum biochemical panel and do not by themselves establish comprehensive hepatic or renal dysfunction.

### 3.2 Descriptive Histological Observations of Liver and Kidney Tissues After DBDPE Exposure and Res Pretreatment

As shown in the representative images in Figure 2A, liver sections from the Control and Res groups displayed regularly arranged hepatic architecture. The displayed DBDPE fields contained hepatocyte vacuolization, areas with a necrosis‐like appearance, cell aggregates and altered tissue arrangement; these are morphological descriptions without cell‐type‐specific confirmation. Oil Red O staining showed more prominent lipid droplets in representative sections from the DBDPE group (Figure 2B). In the Res pretreatment groups, these features appeared less prominent in some representative fields, including those from the H‐Res group.

**FIGURE 2:**
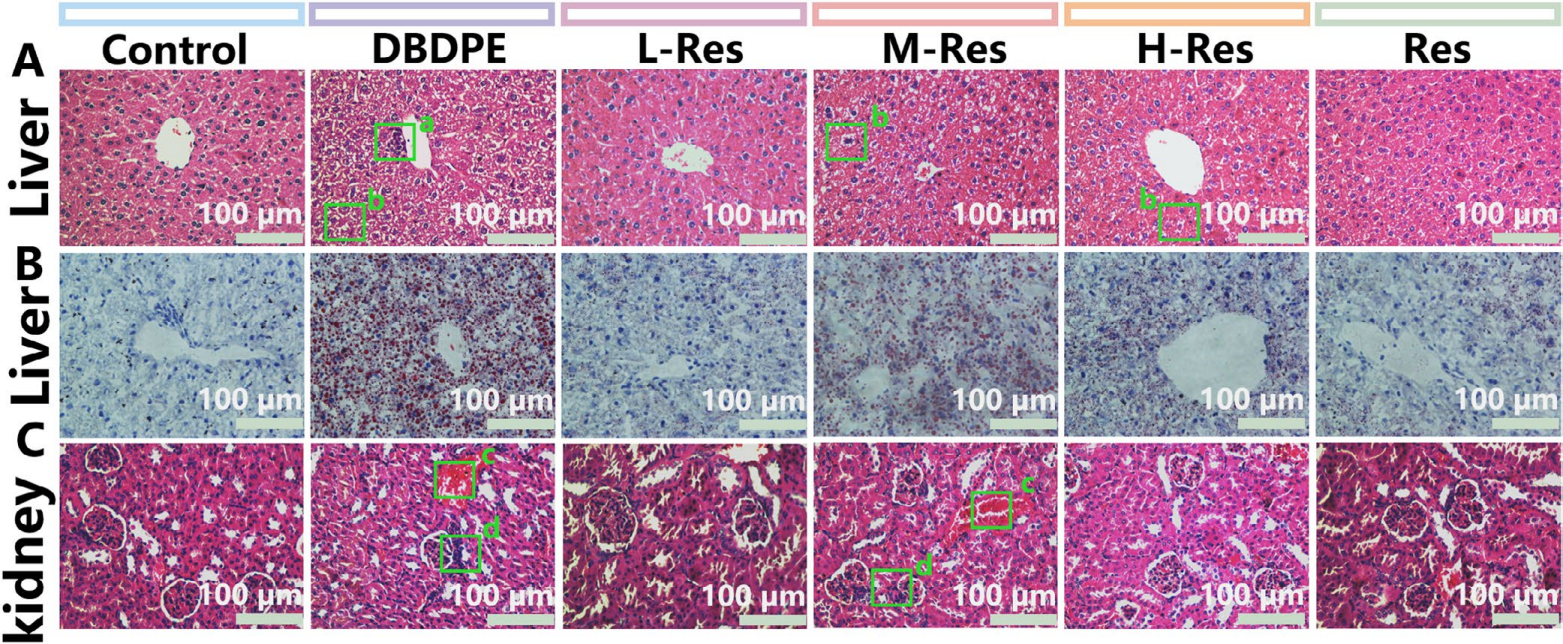
Representative histological observations in liver and kidney tissues after DBDPE exposure and Res pretreatment. (A) Representative H&E‐stained liver tissue: a, cell aggregation; b, cytoplasmic vacuolization. (B) Representative Oil Red O‐stained liver tissue showing lipid droplets in hepatocytes. (C) Representative H&E‐stained kidney tissue: c, cell aggregation; d, congestion. Scale bar = 100 μm.

Representative renal H&E sections from the Control and Res groups displayed relatively preserved glomerular and tubular structures. Sections from the DBDPE group showed renal tubular epithelial degeneration and desquamation, periglomerular cell aggregation, vasodilation, and congestion. In representative sections from the Res pretreatment groups, some of these features appeared less prominent, particularly in the M‐Res and H‐Res groups (Figure 2C). These observations are descriptive; no histopathological scoring was performed, and no quantitative conclusion regarding lesion severity or treatment efficacy was drawn from the sections.

### 3.3 Res Pretreatment and Redox‐Related Changes in Serum, Liver, and Kidney Tissues

As shown in Figure 3A, the DBDPE group differed from the Control group in several serum redox‐related endpoints. Compared with the Control group, serum MDA increased from 8.05 to 10.61 nmol/mL and TNOS increased from 12.23 to 14.81 U/mL, whereas GSH‐ST, GSH‐Px, and SOD decreased from 20.53 to 18.50 U/mL, 428.19 to 368.99 U/mL, and 272.02 to 185.50 U/mL, respectively. Values in the Res pretreatment groups differed from those in the DBDPE group to varying degrees. Serum MDA was 6.54 nmol/mL in the L‐Res group, and GSH‐ST, SOD and TNOS were 24.42 U/mL, 243.31 U/mL and 11.50 U/mL, respectively, in the H‐Res group.

**FIGURE 3:**
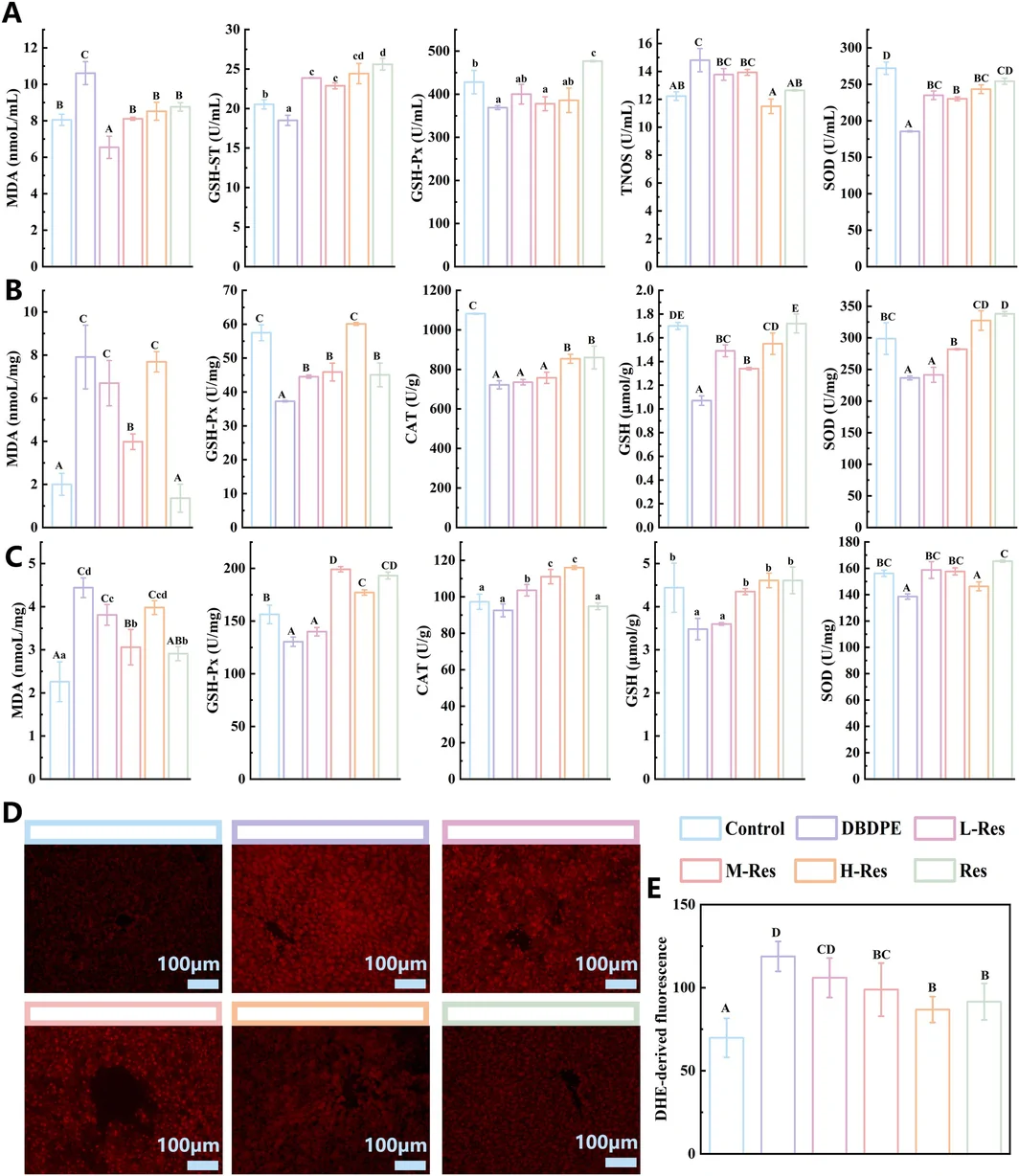
Redox‐related measurements in serum, liver, and kidney tissues after DBDPE exposure and Res pretreatment. (A) Serum markers of oxidative stress, including MDA, GSH‐ST, GSH‐Px, SOD, and TNOS (*n* = 3). (B) Liver tissue markers of oxidative stress, including MDA, GSH‐Px, CAT, GSH, and SOD (*n* = 3). (C) Kidney tissue markers of oxidative stress, including MDA, GSH‐Px, CAT, GSH, and SOD (*n* = 3). (D) Representative DHE fluorescence image in liver tissue. (E) Quantitative analysis of DHE‐derived fluorescence intensity in liver tissue (*n* = 3). Scale bar = 100 μm.

Hepatic redox‐related endpoints differed between the DBDPE and Control groups (Figure 3B): hepatic MDA was higher in the DBDPE group (7.91 vs. 2.00 nmol/mg), whereas GSH‐Px, CAT, GSH and SOD were lower (37.26 vs. 57.49 U/mg, 721.83 vs. 1082.02 U/g, 1.07 vs. 1.70 μmol/g, and 236.55 vs. 298.84 U/mg, respectively). Hepatic MDA was 3.98 nmol/mg in the M‐Res group, and GSH‐Px, CAT, GSH and SOD values in the H‐Res group were 60.11 U/mg, 853.90 U/g, 1.55 μmol/g and 327.48 U/mg, respectively. These group differences describe lower MDA and higher antioxidant values in some Res pretreatment groups than in the DBDPE group.

Renal redox‐related endpoints also differed between the DBDPE and Control groups (Figure 3C): renal MDA was higher in the DBDPE group (4.44 vs. 2.26 nmol/mg), whereas GSH‐Px, CAT, GSH and SOD were lower (130.40 vs. 156.34 U/mg, 92.58 vs. 97.28 U/g, 3.48 vs. 4.44 μmol/g, and 138.60 vs. 156.19 U/mg, respectively). Renal MDA was 3.81, 3.06 and 3.98 nmol/mg in the L‐Res, M‐Res and H‐Res groups, respectively. GSH‐Px was 199.18 and 177.14 U/mg in the M‐Res and H‐Res groups, with GSH values of 4.35 and 4.61 μmol/g, respectively, and CAT activity was 115.99 U/g in the H‐Res group. These differences are consistent with lower measured renal MDA in several Res pretreatment groups, but the assay panel does not identify a specific protective mechanism.

DHE staining showed that DBDPE exposure was associated with markedly higher DHE‐derived fluorescence in liver tissue, whereas fluorescence intensity was lower in the Res pretreatment groups (Figure 3D,E). Together, these data describe group differences in several systemic and tissue redox‐related endpoints.

### 3.4 Res Pretreatment Was Associated With Changes in Antioxidant‐Response‐Related and Ferroptosis‐Associated Transcripts

Oxidative stress‐related transcriptional responses. RT‐qPCR analysis showed that DBDPE exposure was associated with lower hepatic and renal expression of the selected transcripts Pik3r1, Nfe2l2, Sod2, Gpx1 and Cat than in the Control group (*p* < 0.05 or *p* < 0.01). In liver tissue, Sod2 and Cat expression decreased from 1.00 ± 0.08 and 1.00 ± 0.22 in the Control group to 0.29 ± 0.04 and 0.24 ± 0.06 in the DBDPE group, respectively. In the M‐Res group, the corresponding values were 11.11 ± 0.38 and 9.58 ± 0.19 (Figure 4A). In kidney tissue, Nfe2l2 and Cat expression decreased from 1.00 ± 0.03 and 1.00 ± 0.10 to 0.47 ± 0.01 and 0.33 ± 0.07, respectively, whereas their expression in the H‐Res group was 3.55 ± 0.09 and 1.90 ± 0.56 (Figure 4B). The remaining antioxidant‐related transcripts showed a similar general response, although the magnitude varied among Res doses and between tissues. Notably, the response was not monotonic across the three Res doses. These findings describe tissue‐ and dose‐dependent changes in the measured transcripts; they do not establish coordinated pathway activity or PI3K/Nrf2 activation.

**FIGURE 4:**
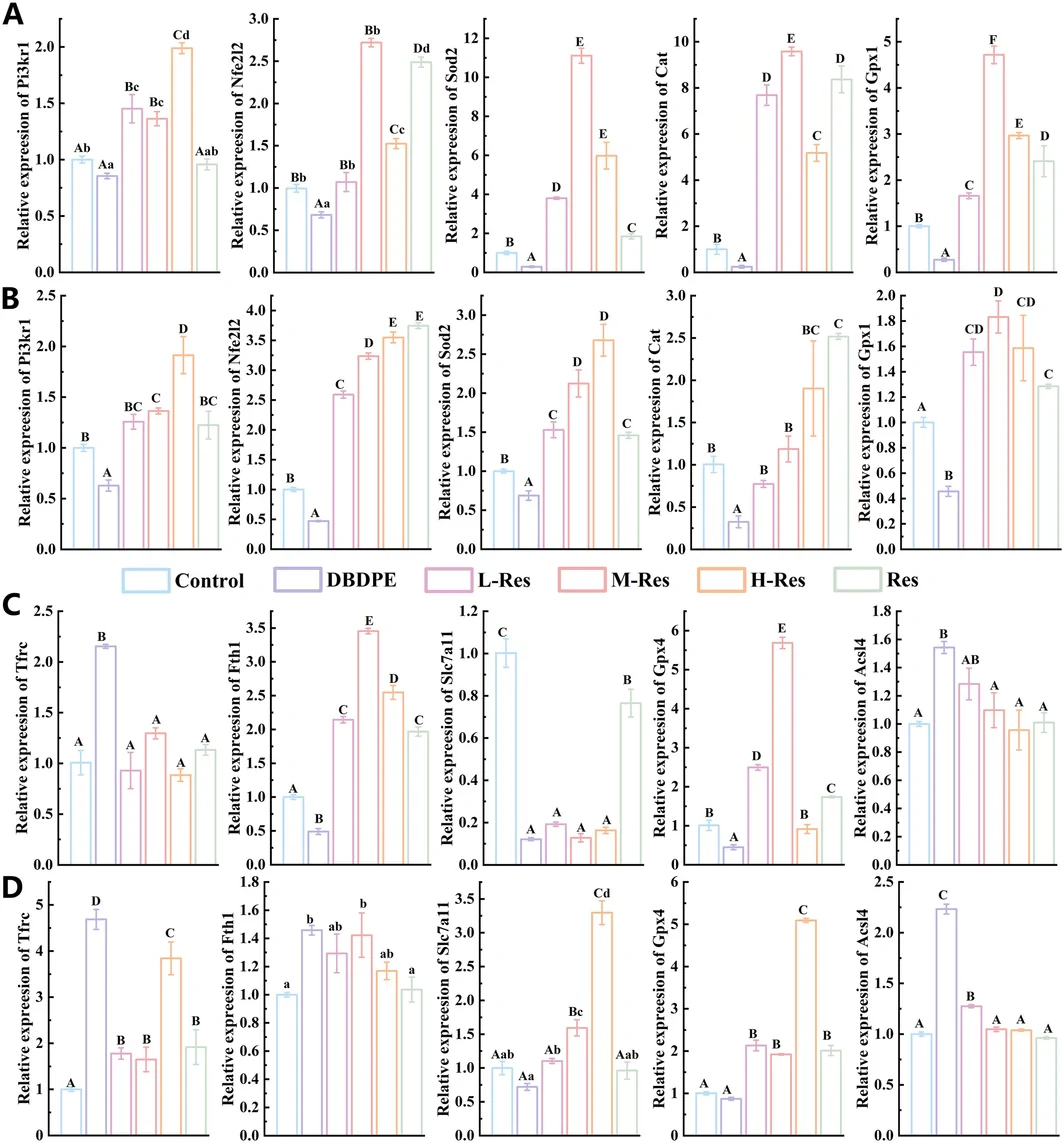
Treatment‐associated changes in selected antioxidant‐related and ferroptosis‐associated transcript measurements in liver and kidney tissues (*n* = 3). (A) mRNA expression of selected genes in liver tissue and (B) kidney tissue, including Pik3r1, Nfe2l2, Sod2, Cat, and Gpx1. (C) mRNA expression of ferroptosis‐associated genes in liver tissue and (D) kidney tissue, including Tfrc, Fth1, Slc7a11, Gpx4, and Acsl4.

Ferroptosis‐associated transcriptional changes. DBDPE exposure was also associated with significant, tissue‐dependent changes in ferroptosis‐associated transcripts (*p* < 0.05 or *p* < 0.01). In liver tissue, Tfrc and Acsl4 expression increased from 1.01 ± 0.12 and 1.00 ± 0.02 in the Control group to 2.15 ± 0.02 and 1.54 ± 0.04 in the DBDPE group, whereas Slc7a11 and Gpx4 decreased from 1.00 ± 0.07 and 1.01 ± 0.13 to 0.12 ± 0.01 and 0.45 ± 0.06, respectively (Figure 4C). H‐Res pretreatment was associated with lower Tfrc and Acsl4 expression (0.88 ± 0.06 and 0.96 ± 0.14), while M‐Res was associated with higher Gpx4 expression (5.68 ± 0.14). However, Slc7a11 remained low in all DBDPE‐exposed Res pretreatment groups, and hepatic Fth1 showed a divergent response. In kidney tissue, Tfrc and Acsl4 expression was higher in the DBDPE group than in the Control group (4.69 ± 0.22 vs. 1.00 ± 0.05 and 2.23 ± 0.05 vs. 1.00 ± 0.02, respectively; Figure 4D). In the H‐Res group, Slc7a11 and Gpx4 expression was 3.30 ± 0.18 and 5.09 ± 0.05, respectively, and Acsl4 expression was 1.04 ± 0.01. In contrast, the reduction in renal Tfrc was more apparent in the L‐Res and M‐Res groups than in the H‐Res group, further indicating a non‐monotonic response. Overall, the ferroptosis‐associated transcriptional changes were more consistent in kidney than in liver tissue but were not uniform across all markers or Res doses. These results should therefore be interpreted as changes in ferroptosis‐associated transcripts rather than evidence that ferroptosis occurred or was inhibited by Res.

Comparisons between the Control and Res‐alone groups showed that Res administration alone was associated with significant changes in several transcripts. In liver tissue, the Res‐alone group showed higher Nfe2l2, Sod2, Cat, Gpx1, Fth1, and Gpx4 expression and lower Slc7a11 expression than the Control group. In kidney tissue, the Res‐alone group showed higher Nfe2l2, Sod2, Cat, Gpx1, Tfrc, and Gpx4 expression (*p* < 0.05 or *p* < 0.01). These changes show that Res at 100 mg/kg was accompanied by independent transcriptional responses in the absence of DBDPE. Because the changes were marker‐ and tissue‐dependent, they should not be interpreted by themselves as evidence of either benefit or toxicity.

### 3.5 Descriptive Mitochondrial Ultrastructural Observations After DBDPE Exposure and Res Pretreatment

Representative TEM micrographs of liver and kidney tissue from the Control and Res groups showed mitochondria with regular morphology, intact‐appearing double membranes, and clearly visible cristae (Figure 5A,B). In representative micrographs from the DBDPE group, mitochondria displayed irregular morphology, shrinkage or deformation, blurred or locally discontinuous membranes, reduced or fragmented cristae, and increased matrix electron density.

**FIGURE 5:**
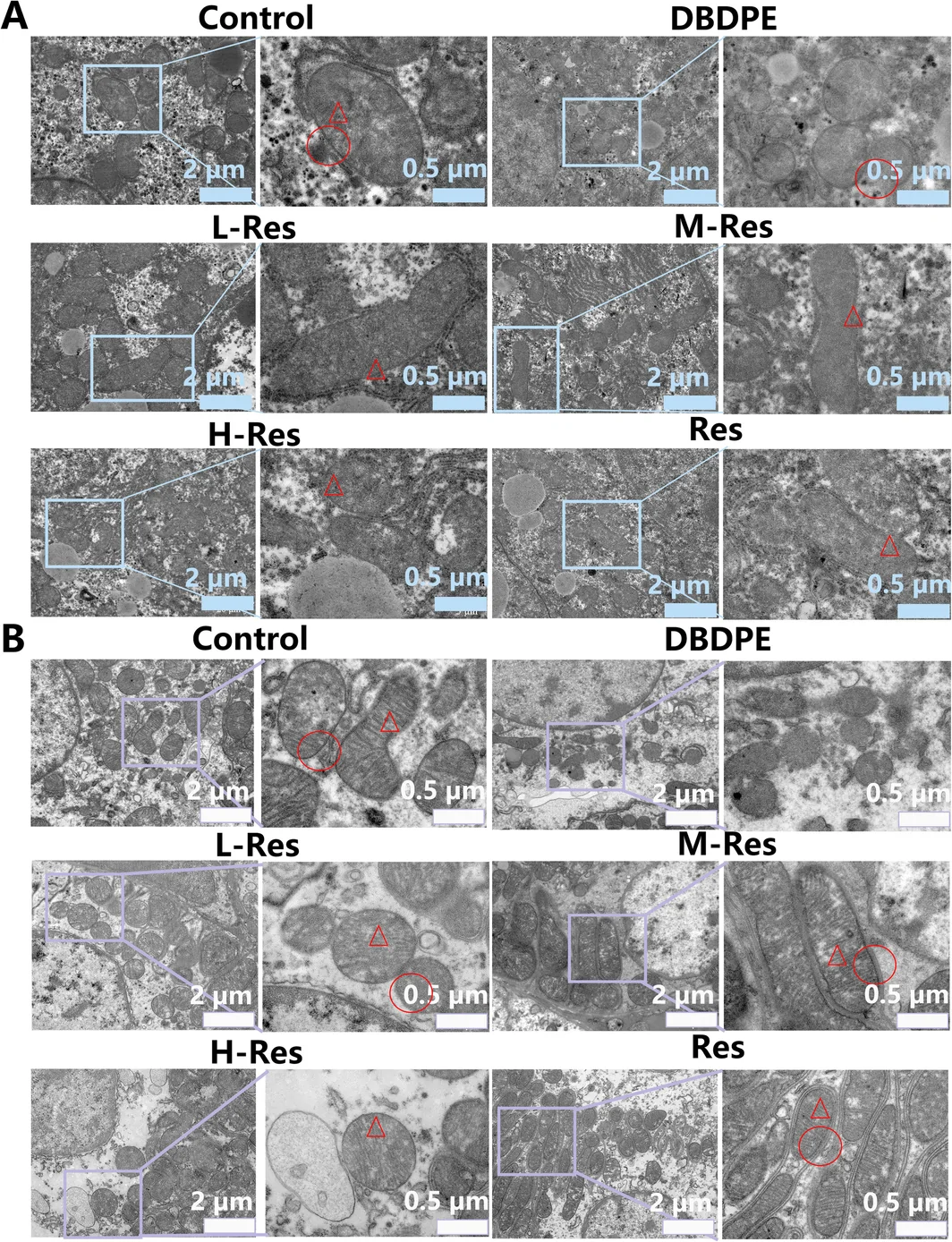
Representative mitochondrial ultrastructure in liver and kidney tissues after DBDPE exposure and Res pretreatment. (A) Representative transmission electron micrographs of liver mitochondria and (B) of kidney mitochondria. Red circles indicate the mitochondrial double membrane and red triangles indicate mitochondrial cristae. Scale bars: Overview fields, 2 μm; enlarged fields, 0.5 μm.

In the displayed fields from the Res pretreatment groups, some mitochondria had more clearly visible membranes or cristae and less conspicuous electron‐density changes than those illustrated for the DBDPE group. These observations are restricted to the representative fields shown; without quantitative morphometry they do not establish the frequency or severity of mitochondrial abnormalities, group‐level differences, or restoration of mitochondrial function.

### 3.6 Res Pretreatment, Assay‐Accessible Iron Readouts and Ferroptosis‐Associated Protein Changes

DBDPE exposure was accompanied by higher assay‐accessible iron readouts in serum, liver and kidney tissues (reported as Fe^2+^; see Section 2.8). Compared with the Control group, serum Fe^2+^ levels increased from 57.41 ± 0.79 to 91.76 ± 1.37 μmol/L in the DBDPE group. Liver Fe^2+^ levels increased from 2.01 ± 0.03 to 3.44 ± 0.02 μmol/g protein, and kidney Fe^2+^ levels increased from 2.33 ± 0.05 to 4.61 ± 0.04 μmol/g protein (Figure 6A). Values were lower in the Res pretreatment groups than in the DBDPE group across the three sample types.

**FIGURE 6:**
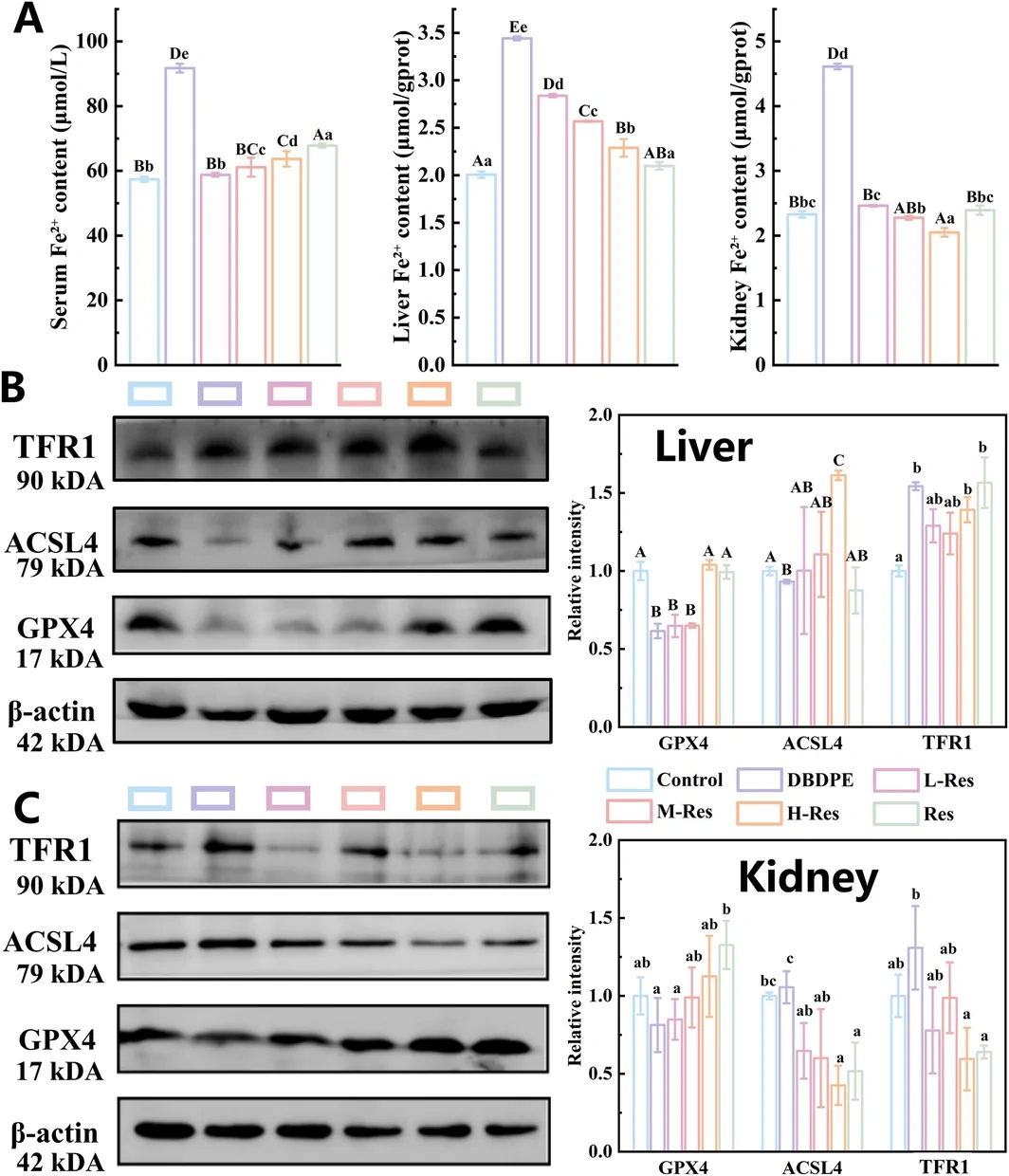
Fe^2+^ Levels and ferroptosis‐associated protein changes after DBDPE exposure and Res pretreatment (*n* = 3). (A) Fe^2+^ levels in serum, liver tissue, and kidney tissue. Serum Fe^2+^ levels are expressed as μmol/L, whereas liver and kidney Fe^2+^ levels are expressed as μmol/g protein. (B) Protein expression and densitometric quantification of GPX4, TFR1, and ACSL4 in liver tissue. (C) Protein expression and densitometric quantification of GPX4, TFR1, and ACSL4 in kidney tissue. TFR1 is encoded by Tfrc. Protein levels were normalized to β‐Actin. Blots are cropped representative images.

Western blot analysis showed treatment‐associated differences in proteins commonly linked to ferroptosis (Figure 6B,C). In liver tissue, DBDPE exposure was associated with lower GPX4 and higher TFR1 abundance; GPX4 abundance was higher in the H‐Res group, and TFR1 abundance was lower in the L‐Res and M‐Res groups than in the DBDPE group. Hepatic ACSL4 did not show the canonical DBDPE‐associated increase, so the liver protein pattern was not uniformly consistent across markers. In kidney tissue, group differences in GPX4, TFR1 and ACSL4 abundance were less consistent across markers, and a uniform protein‐level change was not observed. Together with the Fe^2+^ measurements, these findings describe tissue‐dependent changes in iron levels and ferroptosis‐associated proteins; they do not establish ferroptotic cell death or its inhibition by Res.

## 4 Discussion

This study investigated DBDPE‐associated hepatorenal changes in mice and their relationship to Res pretreatment. Repeated DBDPE exposure was accompanied by selected serum biochemical alterations, descriptive histological features, redox imbalance, higher assay‐accessible iron readouts, mitochondrial ultrastructural abnormalities, and changes in ferroptosis‐associated markers. Several quantitative endpoints differed between the Res pretreatment and DBDPE groups, and representative histological sections also differed among groups. Overall, the results support associations with changes in the measured Pik3r1 and Nfe2l2 transcripts and with ferroptosis‐associated markers, rather than demonstrating activation of a PI3K/Nrf2 pathway or ferroptotic cell death. From a food‐safety perspective, repeated oral exposure provides an experimental route for examining contaminant‐associated effects, but the high‐dose animal model does not represent typical human dietary exposure.

Studies on the in vivo distribution of DBDPE have shown that repeated oral exposure can result in accumulation in tissues such as the liver, suggesting that the liver may be one of its major target organs (Sun et al. 2024; Van Der Schyff et al. 2023). In the present study, DBDPE exposure was accompanied by serum biochemical changes and descriptively observed histological features in liver and kidney tissues. The displayed hepatocyte vacuolization, cell aggregates and lipid droplets provide descriptive morphological context. Without scoring or morphometry, these images do not quantify tissue injury or demonstrate impairment of filtration, reabsorption or lipid metabolism. Previous studies have reported that DBDPE can induce oxidative stress in hepatocytes, decreased mitochondrial membrane potential, and endoplasmic reticulum stress (Jing et al. 2022).

DBDPE exposure was associated with a pattern of redox imbalance in the liver and kidney. In this study, DBDPE exposure was accompanied by higher MDA and DHE‐derived fluorescence and lower levels or activities of several antioxidant markers. Elevated MDA is compatible with increased lipid peroxidation, whereas lower GSH, GSH‐Px, SOD, and CAT values indicate changes in measured antioxidant defenses. These observations are consistent with previous reports of DBDPE‐associated redox changes in L‐02 hepatocytes (Jing et al. 2022). Lower or partially normalized values after Res pretreatment indicate group differences in the measured redox endpoints. Because these markers are not specific to ferroptosis and no temporal or intervention‐based mediation analysis was performed, the results do not establish that oxidative stress preceded ferroptosis or mediated the hepatorenal changes.

The PI3K/Nrf2 signaling axis is an important endogenous defense system against oxidative stress. Nrf2 regulates multiple antioxidant and detoxification‐related genes, thereby enhancing cellular resistance to oxidative damage (Jiang et al. 2024). In this study, RT‐qPCR measured Pik3r1, Nfe2l2, Sod2, Cat, and Gpx1 transcripts. DBDPE exposure and Res pretreatment were associated with group differences in these mRNAs, which are reported individually as changes in selected antioxidant‐related transcripts. These data do not demonstrate PI3K activity, Nrf2 protein abundance or nuclear translocation, direct free‐radical scavenging, or pathway‐mediated protection. Nrf2 is also linked in the literature to GPX4/SLC7A11‐related antioxidant and ferroptosis‐associated defenses (L. Zhang et al. 2024). Previous studies have shown that Res can reduce ROS, MDA, and Fe^2+^ accumulation through Nrf2‐related pathways and enhance protective molecules such as GPX4 and SLC7A11 (Du et al. 2024; Peng et al. 2024). The agreement with prior literature provides biological context, but the present data remain transcript‐level associations and do not establish activity, restoration or causal involvement of a PI3K/Nrf2 pathway.

Several endpoints measured in this study are commonly associated with ferroptosis. These include iron accumulation, enhanced lipid peroxidation, mitochondrial ultrastructural abnormalities, and changes in the GPX4/SLC7A11 defense system (Chen et al. 2024; Zhou et al. 2024). In this study, DBDPE exposure was accompanied by higher assay‐accessible iron readouts, mitochondrial ultrastructural changes, and changes in Tfrc, Gpx4, Slc7a11, Acsl4 and Fth1 transcripts and in selected GPX4, TFR1 and ACSL4 protein measurements. These measurements describe changes in iron handling, lipid‐peroxidation‐related defense, and ferroptosis‐associated markers. Mechanistic evidence from other DBDPE‐related models provides context for interpreting these markers (Shi et al. 2024). In the present study, however, no ferroptosis‐specific inhibitor, rescue experiment, or genetic perturbation was performed, and hepatic ACSL4 protein did not show the canonical increase. The findings are therefore limited to ferroptosis‐associated marker changes.

Group differences after Res pretreatment included lower assay‐accessible iron readouts, changes in GPX4/SLC7A11‐associated transcripts or proteins, and less prominent mitochondrial abnormalities in representative micrographs. GPX4 and SLC7A11 are commonly linked to lipid‐peroxidation defense, whereas TFR1 is related to iron uptake. However, these endpoints were not fully consistent between liver and kidney, including the ACSL4 and FTH1 responses. Accordingly, the findings should be reported as tissue‐dependent changes in ferroptosis‐associated markers, not as proof that Res regulated or inhibited ferroptosis downstream of oxidative stress.

Res has been reported to exert antioxidant, iron homeostasis‐regulating and lipid peroxidation‐inhibiting effects, which may explain its multitarget protective effects against ferroptosis‐associated injury (Peng et al. 2024; Zeng et al. 2024). In the present study, the co‐occurrence of redox‐related, iron‐related, and molecular changes provides biological context but does not establish that Res acted through a coordinated pathway or that ferroptosis mediated the observed effects. Responses across 25, 50, and 100 mg/kg Res were endpoint‐dependent and apparently non‐monotonic: M‐Res showed larger differences for some oxidative‐stress‐related measures and antioxidant transcripts, whereas H‐Res showed larger differences for the iron readouts and some ferroptosis‐associated proteins. Because only three Res doses were tested and no prespecified trend analysis, formal dose–response modeling, or pharmacokinetic measurements were performed, this pattern should not be labeled as hormesis or used to define an optimal dose. Plausible explanations include endpoint‐specific sensitivity, nonlinear absorption or metabolism, saturation of biological responses, concentration‐dependent redox actions, and biological variability; these possibilities were not directly tested. Thus, the current data support only an endpoint‐dependent, apparently non‐monotonic response across the tested doses. The purified‐compound gavage model provides a basis for further animal and mechanistic studies, but it does not support a direct food‐based recommendation.

Res alone was not equivalent to the vehicle control at the transcriptional level. In particular, lower hepatic Slc7a11 expression raises a question about effects on cystine transport and glutathione‐related defense, although transcript abundance alone does not establish transporter function or toxicity. The marker‐ and tissue‐dependent changes in the Res‐alone group preclude a blanket interpretation of all Res‐associated responses as beneficial and require consideration alongside quantitative functional and safety measurements.

This study has several limitations. Histological and mitochondrial observations were based on representative images without semiquantitative scoring or quantitative morphometry and are therefore interpreted descriptively. Normality and homogeneity of variance were checked before the one‐way analysis using Levene's test for homogeneity of variance, but the individual animal values are no longer available and the diagnostic outputs cannot be reproduced. The results for the assays that used three animals per group are therefore reported as exploratory, and the confidence intervals and *p* values reported for the primary outcomes rest on the assumptions of the analysis. Multiplicity was addressed separately for the two primary outcomes, which carry Bonferroni‐adjusted estimates, and for the remaining endpoints, which were compared within each endpoint using Duncan's test. Given the number of endpoints and the small biological sample size for several assays, the findings should be viewed as exploratory and individual significant results interpreted cautiously. The biological sample size for several terminal assays was three per group; these three samples were selected at random after collection from the nine animals in each group, but the small subsample limits the sensitivity of those comparisons.

Only female Kunming mice were studied; sex‐dependent responses were not assessed, and the results should not be generalized to male mice, other strains, or other species. The DBDPE dose was substantially higher than exposure levels expected from concentrations reported in foods, so the findings represent a high‐dose experimental toxicology model and should not be extrapolated directly to realistic dietary exposure. Because only one DBDPE dose was examined, the study cannot establish a DBDPE dose–response relationship, no‐observed‐adverse‐effect level, benchmark dose, margin of exposure, or quantitative dietary risk estimate. Only three Res doses were compared using groupwise analyses, without a prespecified trend test, formal dose–response model, or pharmacokinetic assessment; consequently, the apparent non‐monotonic pattern cannot establish hormesis, a dose–response relationship, or an optimal Res dose. In addition, the study did not include functional ferroptosis rescue, genetic perturbation, pathway‐protein activation, or temporal experiments; the co‐occurring redox, iron‐related, mitochondrial, and molecular changes therefore cannot establish ferroptotic cell death or a causal sequence.

Clinical signs were assessed by visual inspection without a formal scoring system, and estrous‐cycle stage was not monitored or controlled. Food intake was not measured quantitatively, and no conclusion regarding food consumption is drawn. No deaths and no overt abnormal clinical signs were observed in any group; this does not establish the absence of subclinical toxicity or the safety of resveratrol. No separate storage‐stability assessment was performed for the resveratrol formulation, and no analytical verification of the delivered DBDPE concentration, homogeneity or stability was performed. The 18 cages were allocated to fixed positions on the same rack, and each cage contained animals from a single treatment group; cage was not included as a random or fixed effect in the analysis, so the possibility that animals sharing a cage are not fully independent is not accounted for. Assay‐kit details, RNA integrity assessment, primer efficiencies, amplicon sizes, Ct acceptance rules and reference‐gene stability were not fully documented, and Actb stability across treatment groups was not separately validated.

Future studies should address these limitations with lower and multiple DBDPE doses, both sexes, prespecified dose–response analyses, pharmacokinetic or tissue‐exposure measurements, blinded quantitative clinical pathology, functional ferroptosis experiments, protein‐level pathway assays, and systematic clinical monitoring.

## 5 Conclusions

Under the conditions of this 28‐day high‐dose mouse model, repeated oral DBDPE exposure was accompanied by selected serum biochemical alterations and descriptively observed histological features in the liver and kidney. In female mice, these changes co‐occurred with redox imbalance, higher assay‐accessible iron readouts, mitochondrial ultrastructural abnormalities, changes in Pik3r1, Nfe2l2, and selected antioxidant‐related transcripts, and tissue‐dependent changes in ferroptosis‐associated markers. Res pretreatment was associated with lower values for several quantitative endpoints. The findings do not establish ferroptotic cell death, inhibition of ferroptosis, activation of PI3K/Nrf2 signaling, a causal sequence linking oxidative stress to tissue injury, or applicability to typical human dietary exposure. Further work addressing the design and measurement limitations described above is needed before mechanistic or translational conclusions are drawn.

## Author Contributions

**Yutong Ji:** conceptualization, methodology, data curation, writing – original draft, funding acquisition. **Chengzhi Liu:** conceptualization, data curation, writing – review and editing, writing – original draft. **Xianhua Wu:** validation, methodology, data curation. **Mingxian Shi:** validation, supervision, methodology. **Tingjun Hu:** validation, methodology, supervision. **Jiakang He:** validation, supervision, investigation. **Yingyi Wei:** validation, supervision, methodology. **Lei Wang:** funding acquisition, investigation, writing – review and editing. **Meiling Yu:** writing – review and editing, writing – original draft, supervision, project administration, funding acquisition.

## Funding

This work was supported by the Guangxi Natural Science Foundation (Grant number: 2026GXNSFAA00641044), the Open Research Projects of the Guangxi Key Laboratory of Animal Breeding, Disease Control and Prevention (Grant number: ABDC‐b202405), the Innovation Project of Guangxi Graduate Education (Grant number: YCSW2023048), the Guangxi University Natural Science and Technology Innovation Development Multiplier Program Project (Grant number: 2024BZRC013), the Guangxi University Outstanding Doctoral Student Development Program (Grant number: GXUODC2026004), and the Special Research Project for High‐level Talents (Grant number: GXWZY202408). The TOC graphic was created using BioRender.com (https://biorender.com).

## Conflicts of Interest

The authors declare no conflicts of interest.

## Supporting information


**Table S1:** Experimental groups, vehicle controls, and daily oral gavage schedule.
**Table S2:** Primer sequences.
**Table S3:** Primer sequences.

## Data Availability

Data described in this manuscript, including the animal‐level quantitative data and the statistical analysis files, are available from the corresponding author upon reasonable request.
