# Supplementary material for: Resveratrol Pretreatment and Decabromodiphenyl Ethane‐Associated Hepatorenal Changes in a High‐Dose Female Mouse Model: Redox Changes and Ferroptosis‐Associated Markers
Source: Food Sci Nutr. 2026 Oct 16;14(10):e72495. doi: 10.1002/fsn3.72495 (PMC13675606; doi:10.1002/fsn3.72495)
Supplement: Supplementary file 1 [file FSN3-14-e72495-s001.docx]

**Resveratrol Pretreatment and Decabromodiphenyl Ethane-Associated Hepatorenal Changes in a High-Dose Female Mouse Model: Redox Changes and Ferroptosis-Associated Markers**

**Table S1. Experimental groups, vehicle controls, and daily oral gavage schedule**

| Group | Female mice, n | First gavage at 0 h, 0.1 mL per 10 g bw/ mouse | Second gavage at 2 h, 0.1 mL per 10 g bw/ mouse |
| --- | --- | --- | --- |
| Control | 9 | 4% (v/v) DMSO in 0.9% saline | Corn oil |
| DBDPE | 9 | 4% (v/v) DMSO in 0.9% saline | DBDPE, 500 mg/kg bw/day, dispersed in corn oil |
| L-Res | 9 | Res, 25 mg/kg bw/day, formulated in 4% DMSO/0.9% saline | DBDPE, 500 mg/kg bw/day, dispersed in corn oil |
| M-Res | 9 | Res, 50 mg/kg bw/day, formulated in 4% DMSO/0.9% saline | DBDPE, 500 mg/kg bw/day, dispersed in corn oil |
| H-Res | 9 | Res, 100 mg/kg bw/day, formulated in 4% DMSO/0.9% saline | DBDPE, 500 mg/kg bw/day, dispersed in corn oil |
| Res | 9 | Res, 100 mg/kg bw/day, formulated in 4% DMSO/0.9% saline | Corn oil |

Note: All treatments were administered once daily for 28 consecutive days. The DMSO concentration in the first gavage was 4% (v/v) in all groups. Res and DBDPE doses are expressed as nominal doses per kilogram of body weight. DBDPE-containing corn-oil preparations were thoroughly mixed immediately before each gavage administration.

**Table S2 Primer sequences**

| Gene Name | Nucleotide sequence (5' to 3') | Serial number |
| --- | --- | --- |
| *SOD2*-F | CCCAGACCTGCCTTACGACTATG | NM_013671.3 |
| *SOD2*-R | AGCGACCTGAGTTGTAACATCTCC |  |
| *CAT*-F | TCTTGTTCAGTGACCGAGGGATTC | NM_009804.2 |
| *CAT*-R | ATCTTCCTGAGCAAGCCTTCCTG |  |
| *GPX1*-F | GCAATCAGTTCGGACACCAGAATG | NM_001329527.1 |
| *GPX1*-R | CACCAGGTCGGACGTACTTGAG |  |
| *PI3KR1*-F | GGGAGCAGCAACCGAAACAAAG | NM_001024955.2 |
| *PI3KR1*-R | CCACTACGGAGCAGGCATAGC |  |
| *Nfe2l2*-F | TGCCACCGCCAGGACTACAG | NM_001399226.1 |
| *Nfe2l2*-R | ACTTGTACCGCCTCGTCTGGAC |  |
| *Actb*-F | GCTCTGGCTCCTAGCACCAT | NM_007393.5 |
| *Actb*-R | GCCACCGATCCACACAGAGT |  |

**Table S3 Primer sequences**

| Gene Name | Nucleotide sequence (5' to 3') | Serial number |
| --- | --- | --- |
| *ACSL4*-F | TCTGCTTCTGCTGCCCCATTG | NM_001033600.1 |
| *ACSL4*-R | TAACCACCTTCCTGCCAGTCTTTC |  |
| *FTH1*-F | GCGAGGTGGCCGAATCTTCC | NM_010239.2 |
| *FTH1*-R | CCAGTTTGTGCAGTTCCAGTAGTG |  |
| *GPX4*-F | CCGAGTTCCTGGGCTTGTGTG | NM_001037741.4 |
| *GPX4*-R | CCGTCGATGTCCTTGGCTGAG |  |
| *SLC7A11*-F | TGTGTTCGCTGTCTCCAGGTTATTC | NM_011990.2 |
| *SLC7A11*-R | GAAGAGCATCACCATCGTCAGAGG |  |
| *TFRC*-F | GAGTGGCTACCTGGGCTATTGTAAG | NM_001357298.1 |
| *TFRC*-R | CTGACTTGTCTGTCTCCTCCGTTTC |  |
| *Actb-*F | GCTCTGGCTCCTAGCACCAT | NM_007393.5 |
| *Actb*-R | GCCACCGATCCACACAGAGT |  |
